# Negative impact of a health insurer-mandated de-simplification from a single-tablet regimen to a two-tablet regimen

**DOI:** 10.1097/QAD.0000000000003905

**Published:** 2024-04-20

**Authors:** Piter Oosterhof, Matthijs Van Luin, Kees Brinkman, David M. Burger

**Affiliations:** a Department of Clinical Pharmacy, OLVG Hospital, Amsterdam; b Department of Pharmacy, Radboudumc Research Institute for Medical Innovation (RIMI), Radboud University Medical Center, Nijmegen; c Department of Clinical Pharmacy, Meander Medical Center, Amersfoort; d Department of Internal Medicine, Division of Infectious Diseases, OLVG, Amsterdam, The Netherlands.

**Keywords:** antiretroviral therapy, drug regimen simplification, generic drugs, health insurance policies, healthcare costs, HIV treatment strategies, pharmaceutical economics

## Abstract

**Objectives:**

Antiretroviral therapy (ART) accounts for a considerable proportion of HIV care expenses. In June 2021, a Dutch healthcare insurer implemented a mandatory policy to de-simplify branded RPV/TDF/FTC (Eviplera) into a two-tablet regimen containing rilpivirine (Edurant) and generic TDF/FTC as part of cost-saving measures. The objectives of this study were to evaluate the acceptance of this policy, the trends in ART dispensation, and cost developments.

**Design:**

A retrospective database study.

**Methods:**

In this study, medication dispensation data were obtained from the Dutch Foundation for Pharmaceutical Statistics (SFK). This database covers 98% of all medication dispensations from Dutch pharmacies including people with HIV who receive ART. We received pseudonymized data exclusively from individuals insured by the insurer for the years 2020–2022. Costs were calculated using Dutch drug prices for each year.

**Results:**

In June 2021, 128 people with HIV were on branded RPV/TDF/FTC. Following the policy implementation, 59 (46%) had switched to RPV + generic TDF/FTC, but after 1.5 years, only 17 of 128 individuals (13%) used the proposed two-tablet regimen. The other 111/128 used RPV/TDF/FTC with prescriptions for ’medical necessity’ (*n* = 29), switched to RPV/TAF/FTC (*n* = 51), or other ART (*n* = 31). Despite expectations of cost-savings, costs increased from €72 988 in May 2021 to €75 649 in May 2022.

**Conclusion:**

A mandatory switch from an STR to a TTR in people with HIV proved unsuccessful, marked by low acceptance, and increased costs after 1 year. This underscores the necessity of incorporating patient and prescriber involvement in changing medication policies.

## Introduction

The number of people receiving HIV care is steadily increasing, partly because of longer life expectancies [1]. However, these positive developments also lead to financial challenges as individuals require lifelong antiretroviral therapy (ART) [2].

Over the past decade, the use of single-tablet regimens (STRs) has increased significantly, accounting for 37% of ART use in the Netherlands by 2021 [3]. These STRs, often more expensive, contribute to the financial burden within healthcare systems, where medication costs account for 70% of overall expenses [4]. Consequently, controlling these costs without compromising the quality of care is essential to ensure accessible HIV treatment in the future [5,6].

Since late 2017, the introduction of generic forms of frequently used tenofovir disoproxil fumarate/emtricitabine (TDF/FTC) and abacavir/lamivudine (ABC/3TC) [1] has enabled healthcare providers to prescribe medications that are equally effective but at a much lower cost [6]. Several studies have shown that de-simplifying an STR into a two-tablet regimen (TTR) containing the same antiretroviral agents induces serious cost reductions [7–11].

In 2021, a Dutch health insurer implemented a mandatory policy concerning the branded fixed-dose combination of rilpivirine/tenofovir disoproxil fumarate/emtricitabine (RPV/TDF/FTC; Eviplera). Under this policy, the STR was no longer eligible for reimbursement, leading pharmacists to switch patients to a TTR of branded RPV (Edurant) and generic TDF/FTC. The expected savings per patient from this policy were estimated to be approximately €368 per month [12]. However, the effectiveness of this mandatory de-simplification is yet to be determined.

Our study aims to analyze the effects of this health insurer's driven policy to de-simplify treatment from Eviplera to a TTR. During 1.5 years of follow-up, we evaluated the acceptance of this policy, trends in ART dispensation, and cost development.

## Materials and methods

### Data source and period

We used data from the Foundation for Pharmaceutical Statistics (Dutch: Stichting Farmaceutische Kengetallen, SFK), which has been collecting medication usage data in the Netherlands since 1990. This dataset represents over 98% of community and outpatient pharmacies and includes detailed medication dispensation records, pharmacy data, insurance involvement, prescriber details, and patient information while adhering to stringent privacy standards [13].

We selected an observational period from January 2020 to December 2022, spanning approximately 1.5 years before and after policy implementation in June 2021.

### Data structure and editing

The dataset specifically included users of Eviplera and the associated health insurer. We obtained data comprising pseudonymized patient identifiers, geographical (province), demographic (year of birth, sex), and medication-specific information (dispensation date, article number and name, ATC code and description), as well as insurance preference status, dispensed quantities, unit measurements, patent statuses, and pharmacy retail prices.

### Statistical analysis

We conducted an exploratory data analysis approach to assess individual compliance with mandatory medication change, observed trends in ART distribution, and analyzed cost developments on a monthly basis. For cost evaluations, we used Dutch medicine prices, referenced to the corresponding years, enabling an analysis of financial implications [12]. Specifically, we calculated the costs employing the “public pharmacy purchase price” (Apothekers Inkoop Prijs: AIP) as defined by the Dutch Healthcare Authority (NZa) and the Zorginstituut Nederland (ZIN). This price represents the purchase cost paid by pharmacies for the medicines, excluding any negotiated discounts or rebates [14]. To calculate the overall expenditure, we multiplied the AIP for each dispensed ART quantities to calculate costs. This comprehensive method ensured a standardized and transparent approach to assessing the financial implications of the policy change.

### Ethics

Our study obtained ethical approval from the institutional review board of the SFK, ensuring compliance with ethical principles and GDPR guidelines.

## Results

### Acceptance of the policy and alternative antiretroviral therapy dispensation

**Fig. 1:**
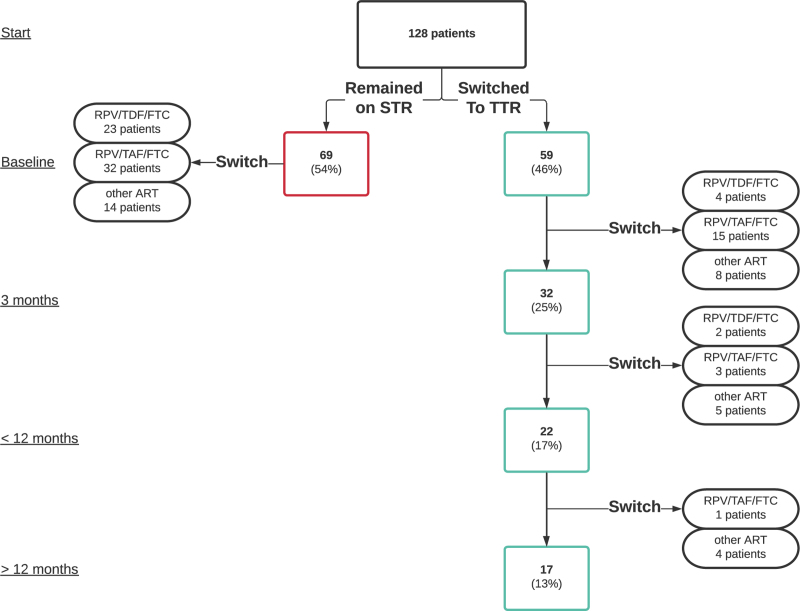
Overview of people with HIV who were eligible for the policy.

Prior to the policy's initiation, 128 people with HIV, insured by the specific health insurer, used Eviplera as an STR (Fig. 1). After the policy was introduced in June 2021, 59 individuals (46%) switched to the proposed TTR. Within the first 3 months, 27 out of the 59 individuals who switched to the TTR discontinued it. An additional 10 discontinued between 3 and 12 months, and a further five stopped within the next 6 months.

**Fig. 2:**
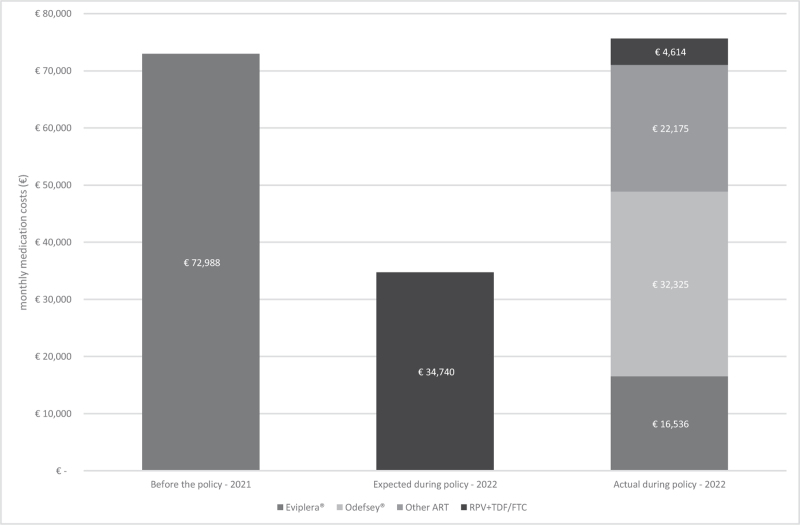
Monthly cost comparison of antiretroviral therapy regimens before and after the policy implementation.

After 18 months of follow-up, 17 out of the 128 individuals (13%) continued using the proposed TTR of branded rilpivirine (Edurant) + generic TDF/FTC, indicating an overall acceptance rate of 13% (Fig. 2). The ART regimens of the remaining 111 individuals at the end of follow-up were 29 (26%) continued on or returned to Eviplera (allowed when prescription is labeled as “ medical necessity” by healthcare provider), 51 (46%) switched to a comparable regimen of STR Odefsey (rilpivirine/tenofovir alafenamide fumarate/emtricitabine -RPV/TAF/FTC), and 31 (28%) switched to other STRs such as bictegravir/tenofovir alafenamide fumarate/emtricitabine (BIC/TAF/FTC) or doravirine/tenofovir disoproxil fumarate/lamivudine (DOR/TDF/3TC).

Costs before the policy were calculated for all individuals using Eviplera, with amounts based on the Pharmacy Purchase Price (AIP). The expected costs in 2022, assuming a policy-driven switch to the generic two-tablet regimen (RPV+TDF/FTC), were also derived using the AIP. The actual costs reflect the varied expenditures on Eviplera, Odefsey, other ART, and RPV+TDF/FTC, indicating the real treatment choices made by individuals after the policy change.

### Cost analysis

Prior to the policy implementation, the monthly costs associated with the use of Eviplera for 128 individuals amounted to €73 000. If all 128 individuals had been effectively switched to the TTR of Edurant + generic TDF/FTC, the costs would have been around €35 000 per month. However, the actual costs following the policy change differed from these projections. According to the ART treatment patterns illustrated in Fig. 1, real-life costs incurred in the postpolicy period amounted to €76 000. Figure 2 shows the cost development and relative contributions of the various ART combinations in this analysis.

## Discussion

Our study showed a remarkable low acceptance rate of 13% following a health insurer-mandated de-simplification from the STR Eviplera to a TTR consisting of Edurant plus generic TDF/FTC. Instead of the intended savings, which could have been more than €38 000 per month, there was an increase of almost €3000 per month (a 4% increase). Notably, the majority shifted to RPV/TAF/FTC (Odefsey) over the proposed TTR.

Worldwide, the increasing costs of ARTs and the introduction of generic alternatives have led to a number of different strategies for cost reductions [15–18]. One of these methods is too de-simplify an STR to a TTR, usually containing a generic NRTI backbone [9,19]. This intervention has been studied by several groups. Giraud *et al.*[9] reported a 45.9% acceptance of de-simplification, with most switchers remaining satisfied postswitch. Krentz *et al.*[19] reported a somewhat higher acceptance rate of 55.1%, achieving substantial cost savings without adverse health outcomes. We recently completed a multicenter study in the Netherlands, demonstrating an initial acceptance rate of 55%, which continued to 45% after one year [20]. All the mentioned studies have in common that prescribers proposed this as a voluntary switch to patients.

The health insurer's policy, which is the subject of the current analysis, differs from the interventions described above in its mandatory aspect, without the involvement of prescribers. Initially, 46% switched to the proposed TTR, which is close to the acceptance rate reported in other studies. However, this high acceptance was enabled by the fact that pharmacies were required to dispense the TTR, as evidenced by a sharp decline in usage within the first 3 months. This decline continued and eventually reached 13%. We suspect that the involuntary nature of the proposed switch contributed to this low acceptance rate.

Interestingly, the health insurance company already announced the mandated switch policy one year earlier, that is, in 2020. At that time, a formal objection was communicated through an alliance of treating physicians, pharmacists, and patient association [21]. As a result, the policy was postponed but not cancelled, and 1 year later, it was nevertheless introduced. In our database, we observed that approximately 42 individuals had already transitioned from Eviplera following the health insurer's announcement in 2020, even before the policy was formally implemented.

When the mandatory switch to the TTR was not accepted, the two most frequent responses were either to switch to Odefsey (46%) or to continue with Eviplera (26%). The STR Odefsey also contains rilpivirine, while TDF is replaced by TAF and cannot be de-simplified, because TAF/FTC is not yet available as a generic backbone. Of note, Odefsey was approximately 10% more expensive than Eviplera in 2022, contributing to an increase in overall treatment expenses [12]. We cannot exclude that in some individuals, renal dysfunction dictated this switch, and not the announced health insurer policy [22].

Although the switch from Eviplera to the TTR is mandatory, within the Dutch healthcare regulations it is still possible for pharmacists to dispense the branded product in case the prescriber notifies “medical necessity” on the prescription. This exception rule is developed for truly exceptional circumstances, such as allergies. Nationwide, this occurs in less than 3% of all medications dispensed in the Netherlands [23]. The high percentage of 26% of persons continuing with Eviplera as “medical necessity” suggests that this exception rule route has been leveraged to prevent switching to the TTR.

Engaging people with HIV and their prescribers is critical for successful implementation of cost-effective treatment changes [24]. This involvement addressed two key issues. First, it could confront potential mistrust in generic antiretrovirals, a concern lessened by numerous studies affirming their noninferiority [7,25,26]. Second, it might challenge the misconception that STRs are superior to TTRs in terms of adherence and treatment outcomes [27,28]. Our multicenter study [20], along with others, demonstrated comparable adherence rates and virological outcomes with those of TTRs [8].

A key strength of our study is the use of a unique dataset from the Netherlands, which provides clear information on the actual medication received by people, including generic dispensations. In addition, such a dataset has never been used before to determine the impact of a health insurer's policy. However, a notable limitation is that this database contains only medication data and lacks additional details, such as treatment outcomes. Moreover, it lacks the capacity to clarify the clinical reasoning behind medication switching. This limitation underscores the complexity of relying solely on a pharmaceutical database for data collection and highlights the need for integrating clinical data into such databases.

Future research should focus on developing ART treatment guidelines that consider factors such as costs, choice between generic and branded antiretrovirals, and real-world treatment outcomes. Implementing such an approach could lead to practical and comprehensive guidelines. Such studies would be important in understanding the impact of these guidelines on patient care, treatment adherence, and overall healthcare costs. Examining the effects of these real-world, evidence-based guidelines could provide new insights into optimizing HIV treatment in different healthcare settings, benefiting patients and the healthcare system. Another aspect of future research should include strategies for successfully implementing therapy changes driven by cost considerations, ensuring that such transitions are both economically beneficial and clinically effective.

## Conclusion

The cost-driven, health-insurance enforced, mandatory switching to a TTR for HIV treatment showed an exceptionally low acceptance rate after 1.5 years in this study. Most people with HIV have switched to more expensive alternative ARTs, leading to unwanted cost increases. These findings highlight the challenges of implementing cost-saving policies and emphasize the importance of including patient and prescriber involvement in HIV care policy implementation.

## Acknowledgements

The authors would like to thank the Foundation for Pharmaceutical Statistics (SFK) for providing this dataset. They would also like to thank Doerine Postma for her contribution to data collection and verification.

All authors contributed to the conceptualization and design of the study. P.O. and D.B. designed the study. P.O. performed formal data analysis and wrote the original draft of the manuscript, including the figures. All authors had full access to all data in the study and had final responsibility for the decision to submit for publication.

The dataset analyzed in this study is not publicly available but can be made available upon reasonable request by the SFK and Piter Oosterhof.

No financial support was provided.

### Conflicts of interest

The authors declared no conflicts of interest.
